# Advances in Therapies to Treat Neonatal Hypoxic-Ischemic Encephalopathy

**DOI:** 10.3390/jcm12206653

**Published:** 2023-10-20

**Authors:** Amaresh K. Ranjan, Anil Gulati

**Affiliations:** 1 Research and Development, Pharmazz Inc., Willowbrook, IL 60527, USA; 2 Department of Bioengineering, The University of Illinois at Chicago, Chicago, IL 60607, USA; 3 College of Pharmacy, Midwestern University, Downers Grove, IL 60515, USA

**Keywords:** endothelin B receptors, hypoxia, ischemia, neonates, sovateltide, IRL-1620, PMZ 1620, neurogenesis, oxidative stress, stem/progenitor cells, regeneration, perinatal asphyxia, cerebral palsy

## Abstract

Neonatal hypoxic-ischemic encephalopathy (HIE) is a condition that results in brain damage in newborns due to insufficient blood and oxygen supply during or after birth. HIE is a major cause of neurological disability and mortality in newborns, with over one million neonatal deaths occurring annually worldwide. The severity of brain injury and the outcome of HIE depend on several factors, including the cause of oxygen deprivation, brain maturity, regional blood flow, and maternal health conditions. HIE is classified into mild, moderate, and severe categories based on the extent of brain damage and resulting neurological issues. The pathophysiology of HIE involves different phases, including the primary phase, latent phase, secondary phase, and tertiary phase. The primary and secondary phases are characterized by episodes of energy and cell metabolism failures, increased cytotoxicity and apoptosis, and activated microglia and inflammation in the brain. A tertiary phase occurs if the brain injury persists, characterized by reduced neural plasticity and neuronal loss. Understanding the cellular and molecular aspects of the different phases of HIE is crucial for developing new interventions and therapeutics. This review aims to discuss the pathophysiology of HIE, therapeutic hypothermia (TH), the only approved therapy for HIE, ongoing developments of adjuvants for TH, and potential future drugs for HIE.

## 1. Introduction

Neonatal hypoxic-ischemic encephalopathy (HIE) is a condition of fetal or neonatal brain damage due to perinatal asphyxia characterized by diminished supplies of blood (ischemia) and oxygen (hypoxia) in the fetus or infant during or after birth. Various conditions, such as placental abruption, prolapse of the umbilical cord, and uterine rupture, are well-known factors in the onset of perinatal HIE. It is the most common cause of neurological disability and mortality in newborns (>1 million neonatal deaths globally each year) [1]. HIE affects both premature and full-term neonates, with a prevalence of approximately 1–4 per 1000 neonates in developed countries and ~26 per 1000 neonates in developing countries. About 35% of neonates with HIE develop long-term neurodevelopmental sequelae, such as mental retardation, epilepsy, cerebral palsy, and learning disabilities, while 25% die within the first two years of life [2,3]. 

**Figure 1:**
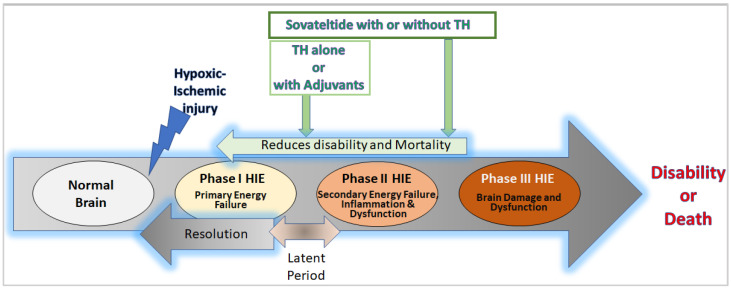
Diagrammatic representation of the phases of hypoxic-ischemic encephalopathy (HIE). After hypoxic-ischemic injury in the normal neonatal brain (white), phase I of HIE (yellow) includes primary energy failure in the neural tissue. The phase I energy failure has a latent period (bi-directional arrow) ranging from 6 h to 15 h (depending upon the severity of the injury), which is a transition period with chances of resolving the issue of primary energy failure due to restoration of blood flow by alternative reperfusion events in the body. Hence, the latent period is also considered the best time for starting a therapy or intervention for treating HIE. If the primary energy failure is unresolved, it leads to phase II of HIE (light orange) with secondary energy failure, overwhelming inflammation, and neural dysfunction. The phase II HIE events are highly devastating and could lead to the phase III HIE (orange) with damaged brain tissue and brain dysfunction, which cause disability or death in HIE patients. At cellular and molecular levels, the primary phase of HIE involves ATP deprivation, abnormal calcium influx, excitotoxicity, and necrosis in neurovascular cells. The secondary phase is marked with oxidative stress following reperfusion, microglial activation, inflammation, excitotoxicity, and cell death (apoptosis/necrosis). The tertiary phase is characterized by decreased neural cell plasticity and a reduced number of neurons (not shown in the diagram). Currently, available interventions for HIE, e.g., TH and adjuvants, are known to act during the latent period (green arrow) and help in reducing the severity of secondary energy failure and would decrease brain damage and dysfunction. However, no evidence of regeneration or repair in the ischemic/hypoxic damaged neural tissue has been reported after treatment with TH and adjuvants. On the other hand, sovateltide (green arrows) has demonstrated mitigation of secondary energy failure and enhancement of neuronal regeneration and repair in the ischemic/hypoxic brain of the HIE rat model and the ischemic stroked brain of the rat and human. Therefore, we anticipate that sovateltide could be developed as a new “First in Class” therapeutic or adjuvant to TH for treating HIE.

The extent of brain injury and the outcome of HIE depend on various factors, including the etiology, extent of hypoxia/ischemia, maturation phase of the brain, regional cerebral blood flow, and maternal diseases/factors affecting the fetus [2]. Based on the extent of brain damage and neurological sequelae determined by clinical examination (Sarnat staging or Thompson score), HIE is categorized into mild, moderate, and severe [4]. The pathophysiology of HIE is complex and has a primary or acute phase followed by a brief “latent phase”, secondary phase, and tertiary phase (Figure 1). 

The primary phase is characterized by primary energy failure (phase duration; minutes), followed by a resolution period or latent phase [2]. In brief, the primary phase starts when supplies of nutrients (glucose) and oxygen are decreased due to reduced blood flow in the brain. In this condition of ischemia/hypoxia, cellular metabolism shifts to an anaerobic type, leading to primary energy failure with decreased ATP production. It affects transcellular transport and accumulates intracellular sodium, water, and calcium, causing membrane depolarization. The depolarization induces excessive glutamate release, which causes excitotoxicity [5]. Anaerobic respiration in this phase increases lactate production and cellular dysfunction or death (apoptosis/necrosis) [6]. However, depending upon the timing of the injury and medical interventions, a partial recovery occurs after 30 to 60 min of the primary phase of the injury, also known as the “latent phase”, and lasts from 1 to 6 h. It is characterized by the recovery of oxidative metabolism, inflammation, and the continuation of apoptotic cascades. This is the most critical phase for utilizing interventions/drugs to control the HIE progression [7]. A secondary phase with another episode of energy failure follows the latent phase and lasts from hours to days. It is characterized by cell metabolism failure, ATP deprivation, an increase in excitotoxicity, cytotoxicity, apoptosis, microglial activation, and inflammation. These events could be lethal or adversely affect the survival and function of neurons in the ischemic/hypoxic neonatal brain, leading to a tertiary phase that lasts from days to years. It is characterized by decreased neural cell plasticity and reduced numbers of neurons. Hence, a better understanding of the different phases of HIE at cellular and molecular levels will help develop new therapeutics/interventions for treating HIE.

This review will describe HIE pathophysiology, therapeutic hypothermia, the only approved therapy for HIE, and potential future therapeutics for HIE. 

## 2. HIE Pathology, Pathophysiology, and Neural Cell Damage

### 2.1. Pathology

The pathology of HIE starts with a reduced supply of blood in the fetal/neonatal brain, which leads to ischemic and hypoxic conditions with nutritional deprivation [8]. The severity of pathological impacts depends upon various factors, including the duration and intensity of interruptions in the blood supply, the age of the fetal/neonatal brain, and the affected brain regions. Depending upon the severity of these impacts, clinical manifestations in the central nervous system vary from one HIE patient to another. Mild HIE is known to cause transient behavioral abnormalities, e.g., poor feeding, irritability, excessive crying, or sleepiness in infants. Also, in the first few days of HIE, the affected neonates/infants seem hyperalert, with brisk deep tendon reflexes and slightly decreased muscle tone. Typically, mild HIE resolves in less than 24 h without any consequences. However, findings from recent years indicate otherwise, showing mild HIE linked with increasingly high adverse outcomes, including brain injury and neurodevelopmental impairment. It has been found that approximately 38–61% of neonates with mild HIE had abnormal brain magnetic resonance imaging (MRI), which is like a moderate to severe HIE population [9,10,11,12]. Moreover, up to 25% of infants with mild HIE had neurodevelopmental impairment [13]. Moderate HIE, also known as “moderately severe HIE”, starts with a sudden deterioration in the neonatal/infantile brain, which has an initial period of well-being or mild hypoxic-ischemic encephalopathy leading to dysfunction, injury, and death of the brain cell, causing seizures. Neonates/infants with moderate HIE are lethargic with diminished deep tendon reflexes and muscle tone (hypotonia). They have poor/no grasping, moro, and sucking reflexes, along with occasional periods of apnea. Seizure in neonates with moderate HIE is typical within the first 24 h of birth; however, full recovery from moderate HIE is possible within 1–2 weeks with a better long-term outcome [14]. Severe HIE is known to cause intense neural dysfunction and damage in the brain following reperfusion injury. It causes stupor or coma in neonates/infants. They may not respond to any physical stimulus, have irregular breathing, and may require ventilatory support. They lack neonatal reflexes (suckling, swallowing, moro, grasping, etc.) and have generalized hypotonia and depressed deep tendon reflexes. Other complications include disturbances of ocular motion, dilation of pupils, and their fixed or poor reaction to light. Typically, seizures are delayed and can be too severe to control using conventional treatments, and their frequency may also increase during the 24–48 h after HIE onset. Neonates/infants who survive severe HIE have hypotonia and feeding difficulties and suffer from multiorgan dysfunction, with prominent functional impairment in the heart, lungs, kidneys, liver, blood, and its vessels (hematological). The heart may have reduced myocardial contractility, dilatation, and tricuspid regurgitation. These impairments cause severe hypotension in HIE neonates/infants. The dysfunction in the lungs leads to pulmonary hypertension and respiratory issues requiring assisted ventilation [15]. Hypoxic-ischemic damage in the kidneys causes renal failure with oliguria, leading to a water and electrolyte imbalance. Liver damage leads to hyperammonemia, coagulopathy, and other gastrointestinal dysfunctions, such as poor peristalsis [16,17]. Hematological abnormalities due to HIE include increased nucleated RBCs, thrombocytopenia, neutropenia or neutrophilia, and coagulopathy [18]. Moreover, a life-threatening rupture of the vein of Galen with hematoma in the posterior cranial fossa of neonates/infants is also possible [19]. Overall, severe HIE can affect both the neuro- as well as cardio-vascular systems and lead to neurological disabilities, cardiorespiratory failure, and death. 

### 2.2. Pathophysiology and Neural Cell Death

Following hypoxic-ischemic injury, complex cascades of events occur in the brain at cellular and biochemical levels, which include oxidative stress, inflammation, excitotoxicity, and cell death (apoptosis/necrosis) [20]. During the early phase of hypoxic/ischemic brain injury, adaptive biochemical processes are started and lead to reduced temperature and increased release of neurotransmitters, e.g., gamma aminobutyric acid transaminase (GABA) in the brain, which reduce oxygen demand to minimize the impact of asphyxia [21]. However, spontaneous or interventional reperfusion in the hypoxic/ischemic brain activates various oxidative enzymes such as cyclooxygenase (COX), xanthine oxidase (XO), and lipoxygenase and leads to increased production of free radicals [22]. In the neonatal brain, the defensive antioxidant system to neutralize free radicals is poorly developed [23]. Consequently, a high amount of free radical production after reperfusion in the HIE brain causes peroxidation of lipids and damages proteins and DNA, leading to inflammation and cell death. Neural cell death is an evolving process and involves primary and secondary energy failures, which set the motion for deleterious biochemical and signaling cascades inducing neural cell dysfunction and death. A. Primary Energy Failure: Primary energy failure starts with reduced cerebral blood flow, leading to a depleted supply of oxygen and glucose in the HIE brain [2]. In this condition, energy (ATP) production is reduced while lactate production is increased [24]. Low ATP levels cause cellular machinery to fail to maintain membrane integrity, Na/K ion pumps, and intracellular Ca^++^ levels. In neurons, the failure of Na/K pumps causes excitotoxicity involving an excessive influx of positively charged Na ions, leading to massive depolarization [25,26]. Upon depolarization, neurons secrete a high amount of glutamate, a prominent excitatory neurotransmitter, which binds to the receptors present in neuronal and glial cells and promotes an additional influx of intracellular Ca^++^ as well as Na^+^. An increased level of intracellular Ca^++^ causes detrimental effects such as cerebral edema, microvascular damage, and cell death (apoptosis or necrosis) [27]. In the mild case of HIE, consisting of less severe hypoxia and ischemia, cells may recover or progress to apoptosis, which leads to programmed cell death (PCD) with cell shrinkage and preservation of the cell membrane with minimal inflammation. On the other hand, in severe hypoxic/ischemic conditions, they undergo accidental cell death (ACD) or necrosis with membrane swelling and rupturing. Rupturing of the membrane causes the release of cellular contents, which promotes associated inflammation [28]. Both apoptosis and necrosis can lead to decreased neural function in the HIE brain; however, the type of cell death in the phase of primary energy failure sets the tone for the next pathological stage of HIE. If neural cell death occurs through ACD or necrosis, the intense inflammatory response and excitotoxicity following reperfusion shorten the latent phase and promote the onset of the secondary energy failure phase. B. Secondary Energy Failure: The secondary energy failure phase starts at 6 to 48 h of hypoxic/ischemic injury. Its onset and progression depend on the extent and severity of the injury. The more severe the injury, the earlier the onset of the secondary phase [29]. Although the exact mechanism of the secondary energy failure remains unknown, necrosis and reperfusion injury in the neonatal brain following primary energy failure are considered key factors. They generate profuse oxidative stress, inflammation, and cell death. When the neonatal brain tissue is re-perfused after ischemia/hypoxia, it fails to adapt to the new aerobic condition. A perfusion injury starts due to the generation of various oxygen and nitrogen reactive species (ROS and RNS). ROS include superoxide anion (O_2_^•−^), hydrogen peroxide (H_2_O_2_), and hydroxyl radical (^•^OH), while RNS include NO and its derivatives. The fetal/neonatal brain is highly susceptible because it is rich in unsaturated fatty acids and iron, has a poorly developed anti-oxidant system, and has a high amount of hydrogen peroxide (H_2_O_2_), which are critical mediators in determining the downstream oxidative stress signaling pathways involved in cell death or repair [30,31]. After perfusion injury, unsaturated fatty acids in the brain break down easily and form oxygen-free radicals, while protein-bound iron is released and forms free iron (Fe^++^), which reacts to H_2_O_2_ and forms hydroxyl radicals (ROS) [32]. The production of these reactive species is catalyzed by specific enzymes present in the cell organelles. They include monoamine oxidase (in mitochondria), cytochrome p450 (in mitochondria, endoplasmic reticulum, and plasma membrane), nitric oxide synthase (NOS) (in peroxisomes), cyclooxygenases (COX) (in the cell membrane), and xanthine oxidase (XO) (in the cytoplasm) [33,34,35]. Following injury, these enzymes in the neonatal brain are activated and generate a burst of reactive species (ROS and RNS) in the HIE brain. Moreover, in a diffusion-controlled reaction, superoxide free radicals (O_2_^•−^) (ROS) and nitric oxide (NO) (RNS) can combine and form a short-lived, highly toxic oxidant “peroxynitrite” [36]. The reaction of peroxynitrite and/or peroxynitrite-derived radicals (for example, carbonate and nitrogen dioxide radicals) with targets results in one- and two-electron oxidations and nitration. Diffusion of peroxynitrite through biomembranes can cause oxidative damage to cellular proteins, lipids, as well as DNA/RNA [37]. In addition to cell damage, the oxidative effects of ROS and RNS also regulate the pro-inflammatory signaling cascade, mitochondrial function, and apoptotic/necrotic signaling pathways [38,39,40,41]. 

In a chronic state of oxidative stress, ROS and RNS activate signaling pathways involved in the production of pro-inflammatory cytokines and chemokines such as IL-6, IL-1β, TNF, and interferons (IFNs), which in turn also induce the production of ROS [42]. Thus, a vicious cycle of oxidative stress and inflammation starts. ROS and RNS act as regulators for inflammatory signaling pathways by oxidizing or nitrosylating amino acid residues of proteins involved in the signaling pathway. For instance, ROS inactivates the protein tyrosine phosphatase (PTP) by oxidizing its cystine residues [43], which results in the perpetual activation of tyrosine kinases (TK) [44]. Consequently, TK-mediated inflammatory and apoptotic signal transduction continues [45]. Moreover, ROS and RNS are also capable of altering the gene expression of various susceptible regulatory transcription factors such as nuclear factor F2-related factor (Nrf2), NF-kβ, activator protein 1 (AP1), HIF-1α, p53, and Forkhead box O (FOXO) [46]. At physiological levels, ROS and RNS activate the DNA binding sites of these transcription factors, which helps in maintaining cellular redox homeostasis; however, in the oxidative stress condition, they over-stimulate the DNA binding site of these transcription factors, resulting in deficient cellular antioxidant defenses and generating high levels of inflammatory mediators [46,47]. 

The inflammatory response is self-regulated in the balanced redox state; nonetheless, it leads to progressive tissue damage under oxidative stress. In the neonatal HIE brain, these inflammatory responses (neuroinflammation) are mediated through glial cells (microglia, oligodendrocytes, and astrocytes) as well as non-glial resident myeloid cells (macrophages and dendritic cells) and peripheral leucocytes [48,49,50,51,52]. These activated neuroinflammatory cells play a significant role in the progression of HIE by inducing neuronal dysfunction and death [53,54]. The activated neuroinflammatory glial cells are also known to secrete ample amounts of glutamate, which induces excitotoxicity and cell death in neuronal cells, as described in the primary energy failure phase [55].

The long-term effects of neuroinflammation and excitotoxicity could prolong the ensuing tertiary phase of HIE, which is characterized by decreased neuronal function and plasticity with increased neuronal cell death [7] (Figure 1). 

## 3. Current Interventions or Therapies for Neonatal HIE

### Therapeutic Hypothermia

Therapeutic hypothermia (TH) in neonates/infants involves lowering the temperature to 33.5 ± 0.5 °C for whole-body cooling and 34.5 ± 0.5 °C for selective head cooling [56]. Nonetheless, successful implementation of cooling protocols requires a multidisciplinary team, including core practitioners who are well-versed in established guidelines [56]. Presumably, it offers neuroprotection by reducing cerebral metabolic demand, decreasing the rate of oxygen consumption, lowering ATP demand, reducing excitatory neurotransmitters, stabilizing the blood-brain barrier, and reducing cerebral edema by decreasing permeability to inflammatory cytokines, free radicals, and thrombin [57,58,59,60]. However, the exact mechanism of action of TH remains unknown. 

Although the mechanism of action of TH remains elusive, TH has proven to be a valuable treatment for infants who have experienced acute perinatal insults such as placental abruption or cord prolapse. This therapy is most effective when administered to term and late preterm infants who are at least 36 weeks gestational age and are less than or equal to 6 h old [61]. To qualify for this treatment, infants must meet specific criteria, including having abnormal cord pH levels, a history of acute perinatal events, low Apgar scores, and signs of encephalopathy [61]. Initiating TH promptly is a critical factor in its effectiveness. In cases where infants need to be transported to specialized units, passive cooling measures such as removing the infant’s hat and blanket and turning off overhead warmers under the guidance of a neonatologist are recommended. It is vital to closely monitor the infant’s temperature during transport, ensuring it remains above 33 °C to prevent excessive cooling [61]. The rewarming process following TH is still debatable regarding the optimal rewarming rate. Primarily, a gradual approach of increasing the infant’s temperature by 0.5 °C every 1 to 2 h over 6 to 12 h is commonly used [61]. However, seizures or a worsening of clinical encephalopathy during rewarming have been reported. In such cases, temporarily stopping the rewarming process and returning to cooling for a 24-h period before resuming the rewarming process is recommended [62].

While therapeutic hypothermia can offer significant benefits, it is not without potential complications [63]. Some of these complications may include bradycardia (a slow heart rate), hypotension (low blood pressure) necessitating the use of inotropic medications, thrombocytopenia (a reduction in blood platelets), and pulmonary hypertension with impaired oxygenation. Additionally, infants with hypoxic-ischemic encephalopathy (HIE) are susceptible to a range of other issues, such as arrhythmias (abnormal heart rhythms), anemia, coagulopathy (blood clotting disorders), and more.

Given the complexity and potential complications associated with therapeutic hypothermia, this treatment must be administered in specialized medical centers staffed by healthcare professionals with expertise in neonatal care and the intricacies of hypothermia therapy. These centers must also be equipped with advanced diagnostic tools like ultrasound, computed tomography (CT), magnetic resonance imaging (MRI), and the ability to perform electroencephalograms (EEGs). Typically, neonatal units categorized as Level 3 and Level 4 meet these requirements and can provide the highest level of care for infants undergoing hypothermia treatment. It is essential to emphasize that the use of ice packs or cool gels by untrained personnel is strongly discouraged, as it can lead to severe hypothermia and should be avoided at all costs [61]. 

Therapeutic hypothermia is a valuable treatment option when administered correctly and carefully monitored to minimize brain injury and improve outcomes for newborns who have experienced perinatal insults. Various trials described below have shown the beneficial effects of TH over conventional care. The Cool-Cap trial tested the efficacy of TH in moderate-to-severe neonatal encephalopathy patients. Selective head cooling with mild systemic hypothermia was induced within 5.5 h of birth. Patients were cooled to a rectal temperature of 34 °C to 35 °C for 72 h. The trial enrolled 234 neonates; 116 of them were subjected to TH treatment, whereas 118 were treated with conventional care [64]. At 18 months of follow-up, the post hoc analysis after controlling for baseline showed improvement in clinical severity outcomes in the TH group. In another multicenter trial on infants with perinatal asphyxia, cooling blankets were used to induce general TH within 6 h of birth. The esophageal temperature was maintained at 33.5 °C for 72 h in 102 patients. The treatment was completed with a slow rewarming. In the control group, 106 infants were assigned [65]. Assessment of these patients at 18 and 22 months of age revealed that patients in the treatment group (TH) had significantly reduced adverse outcomes (i.e., death or disability) (44% vs. 62%; risk ratio, 0.72; 95% CI, 0.54–0.95; *p* = 0.01). The long-term effects of TH on death or IQ score in 97 infants were assessed at 6 to 7 years of age and compared to those of the control group (93 infants). Death or IQ score less than 70 was observed in 47% of TH patients compared to 62% of control infants (*p* = 0.06); 28% of TH patients died compared to 44% in control (*p* = 0.04); death or severe disability occurred in 41% of TH patients compared to 60% in control (*p* = 0.03) [66]. The TOBY (whole body hypothermia) trial was carried out on term infants with moderate to severe perinatal asphyxia. Infants allocated to “intensive care plus total body cooling for 72 h” were compared with those allocated to “intensive care without cooling”. Gel packs were used to achieve the target temperature of 33–34 °C for 72 h in 163 patients. The control group had 162 patients. Whole-body TH was seen as effective and increased survival rate without neurologic sequelae in infants at 18-month follow-up (relative risk [RR], 1.57; 95% CI, 1.16–2.12; *p* = 0.03). Moreover, the incidence of cerebral palsy was lower in the TH group [67]. These trials—NICHD, neo.nEURO.network Trial, the China Study Group, and ICE trials—all showed either an overall benefit of TH for HIE or benefits within subgroups of the trial. All the trials had death and/or disability as a primary composite endpoint. A meta-analysis of the TH trials has shown the effect of TH in significantly reducing the risk for death or moderate to severe neurological disability (n = 249) compared with that of the control group (n = 284) (RR, 0.76; 95% CI, 0.65–0.88). TH was associated with cardiac arrhythmia and thrombocytopenia; nevertheless, they were clinically benign [68]. However, in a recent randomized controlled trial known as HELIX (NCT02387385), conducted in low- and middle-income countries in South Asia, including India, Sri Lanka, and Bangladesh, the use of therapeutic hypothermia (TH) for treating moderate or severe neonatal encephalopathy showed poor outcomes. The study’s results indicated that TH did not effectively reduce brain injury or lead to improvements in disability and mortality outcomes. In fact, the study observed a higher number of deaths in the TH group compared to the control group. The data from the study indicated that a higher percentage of neonates in the therapeutic hypothermia (TH) group (16%) experienced severe thrombocytopenia compared to the control group (7%). This condition could potentially lead to coagulopathy and a series of adverse events in these neonates [69]. Indeed, further optimization of therapeutic hypothermia (TH) in these countries is crucial, especially considering the higher susceptibility to thrombocytopenia observed in women in these regions during pregnancy [70]. Hence, tailoring TH protocols to the population’s specific needs and characteristics can lead to better outcomes and minimize the risk of complications like thrombocytopenia and cardiac arrhythmia. Thus, overall TH clinical trials have demonstrated a significant reduction in death and disability in HIE patients at 18 months of follow-up [71]. However, 40–50% of infants treated with TH still die or have neurological disabilities [72]. This is a matter of concern and has been raised in several workshops and panel experts’ meetings organized by Eunice Kennedy Shriver NICHD, where the efficacy of TH and knowledge gaps were reviewed, and suggestions for research priorities as well as the development of new TH adjuvants aiming to decrease HIE-associated disability and mortality were made [72] because induced hypothermia is known to have certain side effects that can harm neurons [69]. Several adjunctive drugs and interventions are commonly used to minimize the side effects of HIE patients undergoing therapeutic hypothermia (TH). These include morphine, antiepileptic drugs, and interventions for managing blood carbon dioxide levels, avoiding hyperoxia, maintaining blood glucose levels, treating hyperbilirubinemia, and minimizing stimulation [61]. 

Morphine reduces cortisol and noradrenaline, which could help protect neurons [73]. However, its effects on human newborns during hypothermia are uncertain. Neonatal seizures are common in HIE and are suspected to be an independent cause of brain injury, so using antiepileptic drugs as adjuncts to TH is common. Monitoring antiepileptic drug levels is crucial, especially if redosing is necessary. It is important to note that hypothermia can prolong the clearance of such drugs in newborns with HIE [74], so careful attention is required when using these adjunctive treatments. 

To further improve the TH outcomes, preclinical testing of several drugs in a rat model (Vannucci rat model) of HIE has been carried out and identified as potential therapeutics for treating HIE [75]. Unfortunately, none of them proved effective in clinical trials until now. Various adjuvants, including inhaled xenon, erythropoietin, darbepoetin, topiramate, allopurinol, stem cells, and sovateltide, are under trial or completed trial. The following sections will describe some prominent adjuvants and their trials in human patients. 

## 4. Therapies under Development for Neonatal HIE

### 4.1. Therapies under Development with Clinical Study Results

#### 4.1.1. Xenon

Xenon is an odorless noble gas commonly used as an inhalation anesthetic in adults. Xenon has a rapid onset of action via inhalation and exerts anticonvulsant and electroencephalographic (EEG) suppressant effects in HIE neonates [76]. Several preclinical studies have shown its neuroprotective potential at subanesthetic concentrations of 50% xenon [76,77]. Xenon could significantly reduce brain injury in HIE animal models and had an additive neuroprotective effect when combined with TH immediately after the insult [76]. Other studies have also shown its neuroprotective role when administered before, during, and after an HIE [78]. Various concentrations ranging from 40% (Xe40%) to 70% (Xe70%) have been used; however, the optimal timing, dose, and duration of xenon treatment have not yet been optimized. When compared, Xe70% was seen as more neuroprotective than Xe50% in preclinical studies; nonetheless, the recommended dose is established at concentration ≤ Xe50%, which could induce sedation and allow substantial oxygen administration but avoid respiratory depression [76]. At the molecular level, xenon acts as an NMDA receptor antagonist and may prevent glutamate-mediated excitotoxicity [79]. It is known to interact with phenylalanine and bind to the glycine residue of the receptor [80,81]. Neuroprotective properties of xenon have been proven in cell culture [82] as well as in rodent and porcine models of hypoxia-ischemia [83,84,85,86,87,88]. Xenon is also known to activate ATP-sensitive potassium (K_ATP_) channels and two-pore domain potassium [K(2P)] channels, which play roles in neuroprotection [89,90]. Moreover, inhibition of the calcium/calmodulin-dependent protein kinase II [91], activation of the antiapoptotic effectors Bcl-XL and Bcl-2, hypoxia-inducible factor 1 alpha (HIF-1 alpha) erythropoietin, vascular endothelial growth factor, and glucose transporter 1 protein are promoted by xenon [92]. Xenon is believed to exert most of the neuroprotection in the early and late phases of reperfusion injury through inhibition of NMDA receptors and reduction of apoptotic cell death. It acts on the NMDA receptor but lacks dopamine-releasing properties and hence does not increase neuroapoptosis [88,93]. However, the major drawbacks of xenon treatment are its difficulty in use in clinical settings because of its scarcity (0.0087 ppm in air), high costs, and need for closed-circuit delivery (including cuffed tubes) and recycling systems [76]. 

Clinical Trial in HIE Patients: A proof-of-concept randomized controlled trial named “Total Body hypothermia plus Xenon (TOBY-Xe)” (NCT00934700) was conducted at four neonatal intensive care units (NICUs) in the UK from 2012 to 2014 [94]. Ninety-two infants born with evidence of peripartum hypoxia-ischemia were enrolled in the trial. They had moderate to severe encephalopathy and an indication of seizures. Encephalopathy and seizures in neonates were confirmed with an Apgar score ≤ 5 at 10 min after birth and amplitude-integrated EEG, respectively. Infants were randomized, and forty-six were cooled to a target rectal temperature of 33.5 °C and treated with 30% xenon for 24 h. Xenon was inhaled using an uncuffed endotracheal tube and a re-circulator. Infants in the control group were cooled and had a similar SOC to infants in the xenon group. Whole-body cooling was started within six hours of birth and continued for 72 h. A reduction in the lactate-to-N-acetyl aspartate ratio in the thalamus and preservation of fractional anisotropy in the posterior limb of the internal capsule within 15 days of birth were the primary outcomes to be assessed. The trial outcomes showed no significant difference in the lactate-to-N-acetyl aspartate ratio in the thalamus or fractional anisotropy values in the posterior limb of the internal capsule between the xenon and control groups. The mean ratio of lactate to N-acetyl aspartate was 1.09 (95% CI 0.90 to 1.32), and the mean difference in the fractional anisotropy was −0.01 (95% CI −0.03 to 0.02). The mortality did not decrease in the xenon group; 20% of infants died in the control, while 24% died in the xenon cohort. Thus, the TOBY-Xe trial has shown no significant therapeutic effect of xenon over therapeutic hypothermia alone. 

At present, another trial (CoolXenon3; NCT02071394) is underway where the efficacy of xenon with TH in babies with high-risk brain injury following poor condition at birth is being tested. Neonates are treated with whole-body hypothermia for 72 h, started within 3 h of birth, along with 50% xenon inhalation for 18 h, started within 5 h of birth. At 18 months of age, death or moderate to severe disability is being evaluated as the primary endpoint of the trial. 

Thus, the safety of xenon to use with TH has been established; however, its efficacy as a TH adjuvant to treat HIE has not been established until now. 

#### 4.1.2. Erythropoietin and Darbepoetin

Erythropoietin (EPO) is a hormone essential for erythropoiesis, produced primarily by the kidneys in mammals. The production of EPO is regulated through hypoxic cellular responses [95]. Typically, it is inversely proportional to oxygen level; for instance, during hypoxia, its production is elevated and provides a critical erythropoietic response to ischemic stress, e.g., during blood loss and at high altitudes [96]. In such a hypoxic condition, EPO gene expression is enhanced through the binding of HIF-2α to the hypoxic response element of the gene in EPO-producing cells [97,98] present in the kidneys or liver. EPO is released into the bloodstream and acts by binding to its receptor, EPOR, which is expressed on erythroid progenitor cells and non-erythroid cells such as neural cells, endothelial cells, and skeletal muscle myoblasts [99]. Its expression level is highest on erythroid progenitors and promotes their survival, growth, and differentiation needed to produce enough mature RBCs. While a feeble level of EPOR is present in non-erythroid cells, nonetheless, it supports the maintenance and repair of several non-hematopoietic organs, including the brain, heart, and skeletal muscle [100]. Hence, increased expression of EPO in hypoxia/ischemia could be protective for these non-erythroid organs; however, it negatively regulates adipogenesis and osteogenesis. 

Darbepoetin is a supersialylated analog of EPO generated by making five amino acid changes (N30, T32, V87, N88, and T90) in EPO. Darbepoetin contains two extra N-linked glycosylation chains than EPO; however, their tertiary structure and biological activity are identical. It also has greater metabolic stability and a lower clearance rate in vivo. The “elimination half-life” of darbepoetin in humans is three times greater than that of EPO (25.3 h versus 8.5 h). Darbepoetin has more stability, a longer half-life, higher biological activity, and decreased receptor affinity than EPO [101].

Overall, EPO and its analog, darbepoetin, mediate responses that are pleiotropic in nature and are involved in various physiological manifestations following hypoxia or ischemia. Therefore, they are being evaluated as a potential therapeutic target for HIE. Some remarkable clinical trials aimed to assess the safety and efficacy of EPO and darbepoetin to treat HIE are as follows:

EPO Phase I Trials

##### NCT00719407: Neonatal Erythropoietin in Asphyxiated Term Newborns

This was a multicenter, open-label, dose-escalation, phase I study on 24 newborns undergoing hypothermia for HIE (33.5 °C for 72 h within 6 h of HI). The study enrolled 24 newborns with HIE and administered different doses of EPO intravenously: 250, 500, 1000, or 2500 U/kg per dose. Up to six doses were given every 48 h. Pharmacokinetic and safety analyses were performed. The study’s results indicated nonlinear pharmacokinetics of EPO but did not accumulate excessively with multiple dosings. The half-life and maximum concentration of EPO increased with higher doses. At 1000 U/kg per dose, EPO produced plasma concentrations that are considered neuroprotective in animal studies. No deaths or serious adverse effects were observed during the study. The study concluded that intravenous EPO administration of 1000 U/kg per dose with hypothermia is well-tolerated and achieves neuroprotective plasma concentrations [102].

##### ISRCTN33604417: Erythropoietin, Magnesium Sulfate, and Hypothermia for Hypoxic-Ischemic Encephalopathy

This study evaluated the feasibility and outcomes of a combination therapy consisting of EPO, magnesium sulfate, and TH in newborns with HIE. Nine patients meeting the criteria for hypothermia therapy were included. The therapy involved EPO (300 U/kg every other day for 2 weeks), magnesium sulfate (250 mg/kg for 3 days), and hypothermia, starting within 6 h of birth. All patients received a continuous dopamine infusion. The results showed that all nine patients completed the therapy without experiencing death, serious adverse events, or changes in vital signs. At hospital discharge, eight patients achieved oral feeding and did not require ventilation support; however, two patients had abnormal MRI findings. Eight patients underwent follow-up evaluations at 18 months of age, with three showing signs of severe neurodevelopmental disability. Based on these findings, the study concluded that EPO, magnesium sulfate, and TH combination therapy are feasible for newborns with HIE [103]. However, long-term follow-up is needed to assess the overall efficacy and impact on developmental outcomes.

EPO Phase I/II or Phase II Trials

##### NCT00808704: Neurological Outcome after Erythropoietin Treatment for Neonatal Encephalopathy

This was an interventional, phase I/II, randomized, parallel, double-blind study. A total of 167 HIE neonates were enrolled and received recombinant human erythropoietin r-hu-EPO (300 U/kg or 500 U/kg) or placebo treatment every second day for 2 weeks. The first dose of EPO was given subcutaneously the first time (within 48 h of delivery) and then intravenously every other day for 2 weeks. HIE patients who received EPO were less likely to die or have moderate to severe disability at 18 months of age (44% vs. 25%, *p* = 0.02) than those who received a placebo [104].

##### NCT00945789: Erythropoietin in Infants with Hypoxic-Ischemic Encephalopathy (HIE)

This prospective case-control phase I/II study was conducted on 45 neonates grouped into a normal healthy group (N = 15), an HIE-EPO group (N = 15), and an HIE-without EPO group (N = 15). At baseline, the HIE groups had higher blood NO concentrations than the healthy group (*p* < 0.001), while initial clinical severity, pathological, and electroencephalographic findings were similar in the HIE groups. Neonates in the HIE-EPO group were injected with a daily dose of 2500 IU/kg of EPO subcutaneously for 5 days. Results of the study showed that the HIE-EPO group had significant improvement in electroencephalographic data (*p* = 0.01) and decreased NO concentrations (*p* < 0.001) compared to the HIE-control group at 2 weeks of age. Nonetheless, MRI findings were similar between these two groups. Moreover, the HIE-EPO group had fewer neurologic (*p* = 0.03) and developmental (*p* = 0.03) abnormalities at 6 months of age [105]. Thus, these results supported the potential of early administration of EPO to protect against encephalopathy in term neonates with HIE.

##### NCT01913340: Neonatal Erythropoietin and Therapeutic Hypothermia Outcomes in Newborn Brain Injury (NEATO)

In this phase II trial, newborns with moderate/severe encephalopathy and perinatal depression were randomly assigned to receive either EPO (1000 U/kg intravenously; n = 24) or a placebo (n = 26) at specific intervals in the first week of life. All infants received hypothermia treatment. The first dose of EPO was administered at the age of ~16.5 h. The study found that the rates of neonatal deaths did not significantly differ between the EPO and placebo groups. MRI scans conducted over an average of 5.1 days showed that EPO-treated infants had lower global brain injury scores than the placebo group. EPO treatment was associated with a lower incidence of moderate/severe brain injury, subcortical injury, and cerebellar injury than the placebo group. At an average age of 12.7 months, EPO-treated infants demonstrated better motor performance than placebo-treated infants. Thus, the study’s results showed the potential role of EPO in neuroprotection and motor function improvement when combined with TH in HIE patients [106]. The investigators also correlated the placental pathology with the brain MRI scores as part of the study. They observed that EPO was associated with a lower global brain injury score and a lower rate of subcortical injury in subjects with no chronic placental abnormality; however, this was not the case for patients with a chronic abnormality in the placenta. Hence, placental pathology could be a significant factor in the EPO treatment response in HIE [106].

##### PMID: 18676557: A Phase I/II Trial of High-Dose Erythropoietin in Extremely Low Birth Weight Infants: Pharmacokinetics and Safety

This phase I/II trial aimed to assess the safety and pharmacokinetics of high-dose recombinant erythropoietin in extremely low birth weight infants. Eligible infants were <24 h old, ≤1000 g birth weight, and ≤28 weeks of gestation, and had an umbilical artery catheter in place. Thirty infants received three intravenous doses of 500, 1000, or 2500 U/kg EPO at 24-h intervals, while 30 concurrent control subjects received SOC without EPO. The results showed that the infants tolerated high-dose recombinant EPO well, with no excess morbidity or mortality compared to the control group. The EPO concentrations in the blood reached neuroprotective levels observed in animal models. The pharmacokinetics of erythropoietin followed a nonlinear pattern, with clearance decreasing as the dosage increased. A steady state was achieved within 24 to 48 h [107]. Based on these findings, early high-dose recombinant erythropoietin appears safe in extremely low birth weight infants and achieves serum levels associated with neuroprotection. However, further studies are needed to evaluate the efficacy of erythropoietin in improving outcomes in this population.

Darbepoetin Phase I/II and Phase II Trials

##### NCT01471015: Darbe Administration in Newborns Undergoing Cooling for Encephalopathy (DANCE)

This was a phase I/phase II study aimed to evaluate the safety and efficacy of darbepoetin, involving thirty infants undergoing TH treatment for neonatal encephalopathy (NE). They were randomized into three groups: placebo, Darbe low dose (2 μg/kg), and Darbe high dose (10 μg/kg). The first dose was administered within 12 h of birth during the hypothermia period, and a second dose was given at 7 days during the normothermia period. Adverse events were recorded for one month, and serum samples were collected to assess Darbe pharmacokinetics. The study results showed that the adverse events in the treated groups were similar to placebo and historical controls. The pharmacokinetics of Darbe showed half-lives (t1/2) ranging from 24 to 35 h, and the area under the curve (AUC) varied depending on the dose and timing of administration. Nonetheless, clearance of Darbe was not significantly different between the doses. The pharmacokinetics findings supported weekly administration of darbe along with TH treatment. The study concluded that a combination of Darbe and hypothermia has a comparable safety profile to a placebo [108].

##### NCT03071861: Mild Encephalopathy in the Newborn Treated with Darbepoetin (MEND)

This was a phase II multicenter, placebo-controlled, randomized feasibility/safety trial for administering one dose of darbepoetin (Darbe) to infants with mild neonatal encephalopathy and >34 weeks of gestational age. Neonates were randomized and administered either Darbe (10 μg/kg IV) or a placebo (normal saline) at less than 24 h of age. A total of 21 infants (9 in the Darbe group and 12 in the placebo group) were enrolled. Clinical and laboratory data were collected, and developmental assessments were conducted using the Bayley Scales of Infant and Toddler Development, Third Edition (Bayley-III) and a neurological examination at 8–12 months of age. Results showed no adverse events in either group. The Bayley-III score at 8–12 months was not significantly different between groups; nonetheless, the cognitive and language scores were five points higher in the Darbe group (median 100, IQR: 95–115) than the placebo group [109]. 

##### NCT04432662: Darbepoetin in Neonatal Encephalopathy Trial (EDEN)

The EDEN trial is an ongoing 2-arm randomized control phase II trial. It aims to examine the physiological effects of Darbe on proton magnetic resonance spectroscopy thalamic N-acetylaspartate (NAA) levels in babies with neonatal encephalopathy undergoing cooling therapy. A total of 150 babies with HIE (aged < 24 h) are planned to be enrolled in the study. They will be randomized to either the “Darbe with TH” or “TH only” treatment groups. Two doses of darbepoetin (10 mcg/kg) will be administered intravenously following cooling therapy. Brain injury will be examined using EEG, magnetic resonance imaging, and spectroscopy at 1 to 2 weeks of age. The neurological outcomes will be measured at the age of 18 to 22 months. The total estimated trial duration is 4 years, with periods for 4 weeks of startup, 24 months of recruitment, 18 months of follow-up, and 5 months of data analysis as well as presentation.

EPO Phase III Trials

##### NCT03079167: Erythropoietin for Hypoxic-Ischaemic Encephalopathy in Newborns (PAEAN)

This study was a multicenter, double-blind, randomized, placebo-controlled trial on 501 infants with moderate or severe HIE born at 36 weeks or more of gestation [110]. They received EPO (1000 U per kilogram of body weight) or saline placebo in conjunction with TH within 26 h after birth and 2, 3, 4, and 7 days after birth. Death or neurodevelopmental impairment incidence was 52.5% in the EPO group and 49.5% in the placebo group (relative risk, 1.03; 95% confidence interval [CI], 0.86 to 1.24; *p* = 0.74). The mean number of serious adverse events per child was higher in the EPO group than in the placebo group (0.86 vs. 0.67; relative risk, 1.26; 95% CI, 1.01 to 1.57). This phase III study demonstrated that EPO administration to newborns undergoing TH for HIE did not result in a lower risk of death or neurodevelopmental impairment than placebo and was associated with a higher rate of serious adverse events (SAEs) [110]. 

##### NCT02811263: High-Dose Erythropoietin for Asphyxia and Encephalopathy (HEAL)

This was a multicenter, double-blind, randomized, placebo-controlled phase III trial. Five hundred infants born at ≥36 weeks of gestation with moderate or severe HIE undergoing TH were randomized and received EPO or placebo treatment on days 1, 2, 3, 4, and 7. Pre-treatment and post-treatment SAEs were compared. In the modified intention-to-treat analysis of 500 infants, 257 received EPO and 243 received placebo [110]. The incidence of death or neurodevelopmental impairment was 52.5% in the EPO group and 49.5% in the placebo group (relative risk, 1.03; 95% confidence interval [CI], 0.86 to 1.24; *p* = 0.74). The mean number of serious adverse events per child was higher in the EPO group than in the placebo group (0.86 vs. 0.67; relative risk, 1.26; 95% CI, 1.01 to 1.57). The administration of EPO to newborns undergoing TH for HIE did not result in a lower risk of death or neurodevelopmental impairment than placebo and was associated with a higher rate of SAE. Post-treatment thrombosis was also noticed more often in the EPO group (n = 6, 2.3%) than in the placebo group (n = 1, 0.4%; aRR, 95% CI: 5.09, 1.32–19.64). Thus, EPO had a small increased risk of major thrombotic events [110]. Further analysis of the results also indicated that EPO did not reduce biomarkers of neuroinflammation or brain injury in infants with HIE; however, assessment of circulating biomarkers (C5a, IL-6, and neuron-specific enolase at baseline; IL-8, tau, and ubiquitin carboxy-terminal hydrolase-L1 at day 4) modestly improved estimation of 2-year outcomes [111].

##### NCT01732146: Efficacy of Erythropoietin to Improve Survival and Neurological Outcome in Hypoxic-Ischemic Encephalopathy (NEUREPO) 

It is a phase III study on 120 HIE patients aimed at evaluating the efficacy of a high dose of EPO to improve survival and neurologic outcome in asphyxiated term newborns undergoing cooling. It is an interventional, phase III, randomized, parallel, triple-masked trial. EPO intravenous injection (5000 U/0.3 mL) 1000 to 1500 U/kg/dose × 3 was given every 24 h, with the first dose within 12 h of delivery of neonates. The placebo group had 0.2 mL of saline solution × 3 given every 24 h, with the first dose within 12 h of delivery.

Overall, the findings from the above-mentioned clinical studies demonstrate that EPO at an intravenous or subcutaneous dose as high as 2500 U/kg body wt. is safe and well tolerated in healthy as well as HIE neonates; however, its efficacy for treatment of HIE and decreasing the neurological damage due to hypoxia/ischemia has not been estimated. A meta-analysis conducted by Razak et al. [108] aimed to evaluate the effect of EPO administration in neonates with perinatal HIE and concluded that it effectively reduces the risk of brain injury, cerebral palsy, and cognitive impairment. However, a recent meta-analysis conducted by Pan et al. showed that using EPO would not increase the risk of adverse events; however, it is not beneficial for reducing death and improving neurological impairment in HIE neonates [112].

The current state of EPO as a potential drug for HIE is highly diabolical; some studies have demonstrated its effectiveness, while others have failed. Hence, further in-depth analysis of the pleiotropic effects of EPO on erythropoiesis and rescue of non-erythroid organs from hypoxic/ischemic damage in HIE neonates should be carried out. Moreover, pre-evaluation of placental pathology would help achieve the study endpoints, as findings of the NEATO study indicated that EPO was associated with a lower global brain injury score and a lower rate of subcortical injury in subjects with no chronic placental abnormality than in patients that had a chronic abnormality in the placenta. 

Darbepoetin is an EPO analog with higher stability; nonetheless, its action is also pleiotropic like EPO and has been found similar in safety and efficacy parameters. Hence, further development of new EPO analogs with higher binding affinity to non-erythroid EPOR is required to protect the non-erythroid organs from hypoxia/ischemia without interfering with the natural hematopoiesis. 

#### 4.1.3. Topiramate

Topiramate (TPM) is an approved anti-epileptic drug with neuroprotective potential. Its mechanism of action includes selectively blocking voltage-dependent sodium channels, enhancing the inhibitory effect of GABA receptors, and antagonizing glutamate receptors [113]. Although TPM is primarily used as an adjuvant drug for other anti-epileptic medications, it has shown promise as a potential treatment for seizures in neonates with HIE. Nevertheless, its use for this purpose is currently off-label, and limited research on TPM’s efficacy and safety in treating neonatal HIE has been conducted. Some of the clinical studies involving TPM are as follows:

##### NCT01765218: Topiramate in Neonates Receiving Whole Body Cooling for Hypoxic-Ischemic Encephalopathy 

This is an ongoing phase I/II, interventional, parallel, double-blind study carried out on 42 HIE patients. HIE patients to be included in this study should be near term (typically ≥34 wks gestation) and be aged <6 h old, have signs of early perinatal depression (either 10 min Apgar score <5, or pH < 7.00 within 60 min of age, or Base Excess < −12 within 60 min of age, or need respiratory support at 10 min of age due to respiratory depression), and have signs of moderate or severe encephalopathy based on clinical examination or amplitude integrated aEEG assessment. Subjects will receive the SOC and TH (whole body cooling for 72 h followed by gradual rewarming) for HIE. In addition to SOC and TH, subjects in the topiramate group will receive 5 mg/kg of topiramate daily enterally for a total of five doses, while patients in the placebo group will receive a placebo identical to topiramate. Seizures will be assessed for a maximum time point of 4 weeks of postnatal age as a primary endpoint. HIE score (up to 4 weeks of age), normalization of aEEG voltages (up to 4 weeks of age), serum S100-beta (up to 7 days of age), MRI score (up to 7 days of age), and developmental outcome on Bayley scales of infant development III (up to 27 months of age) will be assessed as secondary endpoints.

##### NCT01241019: Safety and Efficacy of Topiramate in Neonates with Hypoxic-Ischemic Encephalopathy Treated with Hypothermia (NeoNATI)

This phase II, interventional, parallel, and single-blind (outcomes assessor) study was carried out on 64 HIE patients [114]. Patients included in this study had gestational age > 36 weeks and birth weight > 1800 g with Apgar score < 5 at 10 min or persisting need for resuscitation, including endotracheal intubation or mask ventilation 10 min after birth or acidosis (pH < 7.0, base deficit >−16 mmol/L in umbilical cord blood or arterial, venous, or capillary blood) within 60 min from birth, or abnormal aEEG, or moderate to severe encephalopathy, consisting of altered state of consciousness (irritability, lethargy, stupor, or coma) and >1 of the following signs: (a) hypotonia, (b) abnormal reflexes, including oculomotor or pupil abnormalities, (c) absent or weak suck, (d) clinical seizures. All subjects were treated with therapeutic hypothermia, while patients in the topiramate group were treated as follows: a dose of 10 mg/kg topiramate was administered once a day for the first 3 days of life, for a total of 3 doses per patient on arrival in the NICU, when the cooling began (T0). The efficacy of topiramate was evaluated by assessing the neurological outcome at 6, 12, and 18 months of life as a primary outcome, while the neuroradiological outcome at 3 and 12 months of life was evaluated as a secondary outcome. Results for forty-four patients were reported. Twenty-one neonates (10 with moderate and 11 with severe HIE) were allocated to the topiramate group and 23 (12 with moderate and 11 with severe HIE) to only the hypothermia group (control). Reduction in the prevalence of epilepsy in topiramate-treated newborns was observed; however, no differences were observed for safety or primary or secondary outcomes in the control and topiramate groups. The study concluded that the administration of topiramate to newborns with HIE is safe but does not reduce the combined frequency of mortality and severe neurological disability in HIE patients [114]. 

Studies carried out until now suggest that short-term use of TPM may benefit patients with HIE; nonetheless, further research is needed to prove the potential of TPM in treating HIE.

#### 4.1.4. Glucocorticoids

Glucocorticoids are known to participate in various homeostatic activities such as blood pressure, the immune system, protein and carbohydrate metabolism, and anti-inflammatory action. Recent studies have shown that the administration of glucocorticoids to the neonatal brain through intracerebroventricular injection or intranasally can provide neuroprotection and improve brain damage after hypoxic-ischemic (HI) injury [115]. Hydrocortisone, a type of glucocorticoid, was tested in a prospective, randomized, double-blind, placebo-controlled, single-center phase II/III study (NCT02700828). The study compared the effect of a low dose (0.5 mg/kg) of hydrocortisone vs. placebo on systemic low blood pressure during hypothermia treatment in asphyxiated newborns. Patients were treated with hydrocortisone (4 doses of 0.5 mg/kg/24 h) or placebo along with conventional inotropic therapy until the end of hypothermia treatment (max. 72 h). Results of the study showed that more patients achieved the target of at least a 5-mm Hg increment of the mean arterial pressure in 2 h in the hydrocortisone group compared with the placebo group (94% vs. 58%, *p* = 0.02, intention-to-treat analysis) [116]. The study concluded that hydrocortisone administration effectively raised the mean arterial blood pressure and decreased the inotrope requirement during hypothermia treatment in HIE neonates with volume-resistant hypotension.

Current Status of Neonatal HIE Therapeutics Under Development: Unfortunately, none of the adjuvants that have completed the trial have shown any benefit over TH alone. The common limitation of the adjuvants being tested is that they are effective enough to limit cerebral damage by controlling the secondary phase events of HIE but fail to repair neuronal damage. Hence, newer therapies or adjuvants, which could prevent and repair neuronal damage by promoting neurogenesis and angiogenesis following HIE, need to be developed and evaluated.

### 4.2. Future Therapies (Therapies under Development with Results Awaited)

In addition to the above-mentioned therapeutics, some other drugs that are being tested in humans are melatonin, caffeine, citicoline, metformin, hydrocortisone, RLS-0071, stem cells, and sovateltide. A full list of registered ongoing/future interventional clinical trials for neonatal HIE with the status of patient recruitment as recruiting/not yet recruiting/unknown is provided in Table 1.

#### 4.2.1. Melatonin

Melatonin is widely known for regulating the circadian rhythm and is mainly produced by the pineal gland [117]. In addition to its role in circadian rhythm, it is known to serve as a free radical scavenger, inhibitor of inflammatory cytokines, and stimulant of anti-oxidant enzymes [118]. Therefore, its potential role in interrupting several key components in the pathophysiology of HIE and minimizing neural cell death is being explored. The safety, tolerability, and efficacy of melatonin are currently being examined in a phase I study (NCT02621944) in infants with HIE and treated with TH. A total of 30 subjects will be enrolled in the study. The first cohort of 10 subjects will receive 0.5 mg/kg of melatonin. If that dose proves to be safe, the second cohort of 10 subjects will receive a dose of 3 mg/kg of melatonin. After the safety assessment, the third cohort of 10 subjects will receive a dose of 5 mg/kg of melatonin. The study will assess various parameters, including the serum concentration of melatonin and adverse events. The long-term safety and potential efficacy via developmental follow-up will be performed at 18–22 months. In addition, the effect of melatonin on the inflammatory cascade, oxidative stress, free radical production, and serum biomarkers of brain injury in neonates will be evaluated. The efficacy of melatonin is also being assessed in another randomized, double-blind, placebo-controlled study on 100 neonates with HIE receiving TH (NCT03806816). Five daily enteral doses of melatonin (10 mg/kg) will be given to the neonates. The effect of melatonin will be assessed using aEEG, MRI, and spectroscopy analysis. Moreover, neurodevelopmental outcomes will be assessed using the Bayley Scales III at 6, 12, and 24 months.

#### 4.2.2. Caffeine

Caffeine is a widely consumed, naturally occurring stimulant of the methylxanthine class that acts on adenosine receptors [119]. It has FDA-approved medical uses, such as treating apnea of prematurity and bronchopulmonary dysplasia in premature infants [120]. There is ongoing research on its potential effectiveness in treating infants with HIE and neurocognitive declines in conditions like Alzheimer’s and Parkinson’s disease. Caffeine affects adenosine receptors [121] in the CNS as well as the cardiovascular system, promotes positive inotropic effects in cardiac muscle, and stimulates the release of catecholamines. It also causes vasodilation through the antagonism of vascular adenosine receptors and the release of nitric oxide [122]. Adenosine is also involved in regulating inflammation. Therefore, caffeine’s antagonistic action on adenosine receptors reduces inflammation in certain conditions [123]. The half-life of caffeine in adults is approximately 5 h, but it can be significantly longer in newborns and premature infants (up to 8 h for full-term and 100 h for premature infants) due to differences in metabolism [120]. In animal models of HIE, caffeine helps reduce white matter brain injury [124]. Moreover, in premature neonates, caffeine was found to be effective in reducing acute kidney injury (AKI) [125]. However, the safety and efficacy of caffeine have not been studied in the setting of TH in HIE infants. Currently, two different phase I trials aimed at studying the pharmacokinetics and safety of caffeine in neonates with HIE having TH are being carried out. In one trial (NCT03913221), the neonates will be given a loading dose of caffeine (20 mg/kg IV), followed by two daily doses of either 5 mg/kg IV or 10 mg/kg IV. In another trial (NCT05295784), the first six neonates will receive a single dose of 5 mg/kg, the next six neonates will receive a single dose of 15 mg/kg, and the next six neonates will receive a single dose of 25 mg/kg of caffeine within the first 24 h of life. These studies will assess the safety and tolerability of caffeine, along with its effects on neurodevelopmental functions (NCT03913221), as well as its impact on seizure burden. 

#### 4.2.3. Citicoline

Citicoline is a form of cytidine 5-diphosphocholine (CDP-choline) and plays a crucial role in the synthesis of phosphatidylcholine from choline [126]. When taken orally, citicoline is rapidly absorbed and broken down into cytidine and choline in the gut. In the context of HIE, citicoline shows promise as a potential neuroprotector [127]. Some of the actions of citicoline include inhibiting glutamate accumulation, promoting the regeneration of injured cell membranes, increasing brain plasticity and repair, boosting the levels of useful neurotransmitters like dopamine and acetylcholine, and preventing the formation of free radicals [128]. Overall, citicoline holds potential as a therapeutic option for treating HIE by aiding in the repair and protection of neuronal cells. A phase III randomized, open label, single-group clinical trial, “Role of Citicoline in Treatment of Newborns With Hypoxic-ischemic Encephalopathy (citicoline)”, was registered (NCT03181646) at ClinicalTrials.gov in 2017; however, the current recruitment status is UNKNOWN. In this study, newborns with hypoxic-ischemic encephalopathy grade 2/3 will receive 15 mg per kg of citicoline intravenously until oral feeds are established, along with supportive care.

#### 4.2.4. Metformin

Metformin is a widely used biguanide for managing type 2 diabetes [129]. It is highly permeable to the BBB and has pharmacological activities as an anti-oxidant, anti-inflammatory, anti-apoptotic, and anti-tumor agent, making it a potential drug candidate for CNS diseases [130]. Recent studies have demonstrated its neuroprotective effects in animal models of spinal cord injury and cerebral ischemia/reperfusion injury [131,132]. It is also known to promote neurogenesis, the integrity of the BBB in experimental strokes, and remyelination in neonatal white matter after injury [133,134]. However, its effects on neonatal brain injury induced by hypoxic-ischemic events are still elusive, and further studies are required to understand its neuroprotective effects on HIE. A phase I study is currently underway to study its safety and treatment feasibility in infants with perinatal brain injury (NCT05590676). The study will enroll ≤3-month-old term and preterm patients. A total of 20 patients will be enrolled in the study. Five infants with HIE will be enrolled in the first dose cohort of 20 mg/kg metformin; once complete, another five infants will be enrolled in the second dose cohort of 25 mg/kg metformin. On the other hand, five preterm infants will be enrolled in the first dose cohort of 15 mg/kg metformin, while another five preterm infants will be enrolled in the second dose cohort of 20 mg/kg metformin after completion of the first dose.

#### 4.2.5. Allopurinol

Allopurinol [4-hydroxy-pyrazole(3,4-d) pyrimidine], an inhibitor for xanthine oxidase (XO) [135], has been shown to have protective effects during ischemia. Inhibition of XO leads to reduced production of ROS and oxidative stress [136]; hence, it is suggested as a way to improve cardiovascular health. Some important ROS that could diminish due to XO inhibition include hydroxyl free radicals, hydrogen peroxide, and peroxynitrite, which can damage DNA, proteins, and cells. XO is also known to catabolize purines [137] in some species, including humans. Allopurinol is commonly used to treat various diseases such as gout, vascular injury, inflammation, ischemic heart disease, and heart failure. It is known to easily cross the blood-brain barrier and participate in neural cell protection [138]. It modulates reactive oxygen species (ROS) production, reduces oxidative stress, and decreases pro-inflammatory molecules in the brain after injury. It has been found to inhibit axonal damage and demyelination caused by oxidative stress and proinflammatory cytokines. Studies have shown that allopurinol administration after cerebral hypoxia-ischemia in neonatal rats reduces brain edema and neural cell death [139]. This neuroprotective effect is attributed to its ability to inhibit xanthine oxidase, which leads to reduced purine degradation and increased accumulation of purine metabolites, including adenosine, during hypoxia/ischemia. In a study with newborn piglets, allopurinol treatment during hypoxia resulted in significantly higher levels of adenosine and inosine in the brain tissue, which may potentiate the intrinsic neuroprotective mechanisms. At present, allopurinol’s effect on human patients with HIE is also being assessed. The details of an ongoing clinical study of allopurinol are as follows: NCT03162653: Effect of Allopurinol for Hypoxic-ischemic Brain Injury on Neurocognitive Outcome (ALBINO). This interventional clinical phase III trial aims to evaluate the potential benefits of early administration of allopurinol in newborns with asphyxia and signs of hypoxic-ischemic encephalopathy. The study will compare allopurinol to a placebo (Mannitol) in addition to standard care, focusing on long-term outcomes such as severe neurodevelopmental impairment or death at two years. The trial will enroll an estimated 760 participants and follow a randomized, parallel assignment design with quadruple masking. Allopurinol will be administered in two doses, with the first given as soon as possible after birth and the second given 12 h later for infants undergoing therapeutic hypothermia. The placebo group will receive Mannitol with the same dosing schedule. The primary outcome measure is a composite endpoint of death, severe neurodevelopmental impairment, or survival without severe neurodevelopmental impairment at 24 months. Secondary outcome measures include death or neurodevelopmental impairment, the incidence of death, the incidence of cerebral palsy, the GMFCS-score for motor impairments, the motor-composite-score, the cognitive-composite-score, the language-composite-score, and various dichotomized scores related to motor, cognitive, and language function. The study will analyze the data using statistical tests such as the generalized logit model, the Cochrane–Mantel–Haenszel X² test, and the Wilcoxon–Mann–Whitney test. The inclusion criteria for the study involve term and near-term infants with a history of disturbed labor who exhibit perinatal acidosis or ongoing resuscitation, as well as early clinical signs of potentially evolving encephalopathy, such as altered consciousness, abnormal muscle tone, respiratory difficulties, and abnormal reflexes or movements. The estimated completion date of the study is January 2026.

#### 4.2.6. RLS 0071

RLS-0071 is a peptide with anti-inflammatory activity developed by RealAlta Life Sciences Inc. [140]. It is being studied as a potential treatment for hypoxic-ischemic encephalopathy (HIE) and other rare diseases. The peptide consists of 15 amino acids and inhibits both humoral and cellular inflammation by blocking complement activation, myeloperoxidase (MPO), and neutrophil extracellular traps [140]. A phase II clinical trial (NCT05778188) is being carried out to test the safety, tolerability, pharmacokinetics (PK), and preliminary efficacy in newborns with HIE. It is a two-stage study of 42 participants with moderate or severe HIE undergoing therapeutic hypothermia for 72 h. In stage 1, they will receive either ascending doses of RLS-0071 or a placebo, in addition to SOC. On completion of stage 1, participants will enter stage 2 of the study, which involves long-term observation and follow-up until 24 months.

#### 4.2.7. Stem Cells

Multipotent stem cells are special types of cells that can self-renew and differentiate into different organ/tissue specific cell types [141]. Some of the well-known stem cells, such as mesenchymal stem cells (MSCs), mononuclear cells, oligodendrocyte progenitor cells, neural stem cells, hematopoietic stem cells, endothelial cells, and inducible pluripotent stem cells, have shown promising therapeutic effects in hypoxic-ischemic damage [142]. The beneficial effects of these cells occur at both the cellular and functional levels. Overall, stem cells have the potential to create a favorable environment for tissue regeneration and lead to better functional outcomes following hypoxic-ischemic damage [142]. Moreover, stem cell therapy has shown positive effects following perinatal hypoxic-ischemic insults, and its potential to treat HIE is being tested in various pre-clinical [143] and clinical studies [144]. Some of the prominent clinical studies are described below. 

##### NCT02854579: Neural Progenitor Cell and Paracrine Factors to Treat Hypoxic-Ischemic Encephalopathy 

This interventional clinical trial aims to investigate the use of allogenic neural progenitor cell transplantation and intrathecal injection of paracrine factors of human mesenchymal stem cells in neonates diagnosed with moderate/severe HIE. The study will enroll 120 HIE neonatal patients. They will be randomized into four arms: neural progenitor cell transplantation, paracrine factor injection, a combination of cell and factor, or routine therapy only. The patients will be followed up, and the interventional safety and efficacy will be assessed at 12 and 18 months by evaluating neurodevelopmental outcome, magnetic resonance imaging, electroencephalogram, Bailey scores, Peabody development measure scale, and gross motor function measure. The study’s primary endpoints include the neonatal behavioral neurological assessment and the number of adverse events. The inclusion criteria specify gestational age ≥ 34 weeks and body weight ≥ 2 kg, with evidence of encephalopathy within 6 h of age or abnormal EEG for more than 24 h. The current status of the study is unknown (last update posted on ClinicalTrials.gov: 3 August 2016).

##### NCT01962233: Umbilical Cord-Derived Mesenchymal Stem Cell Therapy in Hypoxic-Ischemic Encephalopathy

This interventional clinical phase I trial aims to assess the safety and therapeutic effects of umbilical cord blood stem cell infusion in patients with hypoxic-ischemic encephalopathy. The study will enroll 10 HIE patients (child, adult, or older adult) who will receive an intravenous infusion of umbilical cord blood stem cells (100–800 million cells) in addition to conventional therapy. The primary outcome measure is the National Institutes of Health Stroke Scale (NIHSS) score, which assesses neurological deficits. The study will evaluate the curative effects at 15 days, 90 days, and 180 days after treatment. Adverse reactions will also be monitored. The status of the study is unknown (last update posted: 14 October 2013).

##### NCT02881970: Neonatal Hypoxic-Ischemic Encephalopathy: Safety and Feasibility Study of a Curative Treatment with Autologous Cord Blood Stem Cells (NEOSTEM)

This is an ongoing phase I/II, interventional, single group assignment, open label study. It is being carried out on 20 term neonates of ≥36 weeks of gestation. Enrolled patients should have a history of the acute perinatal event (e.g., abnormal fetal cardiac rate, cord prolapse, uterine rupture, maternal hemorrhage), blood pH < 7 with base deficit > 12 mmol/L (at birth or within 60 min of age), or a blood pH between 7.01 and 7.15, with additional criteria, a 5 min Apgar score ≤ 5, or a continued need for resuscitation, including endotracheal or mask ventilation at 5 min after birth, signs of encephalopathy within 12 h of age (Sarnat and Sarnat classification, score ≥ 2), abnormal electroencephalogram or aEEG within 12 h of age, and therapeutic hypothermia. The study aims to evaluate the adverse clinical or paraclinical event rates due to stem cell preparation as a primary endpoint and preliminary efficacy as measured by neurodevelopmental function as a secondary endpoint. These assessments will be done up to 2 years of age.

##### NCT04261335: The Clinical Trial of CL2020 Cells for Neonatal Hypoxic-Ischemic Encephalopathy (SHIELD)

This is an interventional, open-label, single-group assignment, dose escalation phase I study to evaluate the safety and tolerability of CL2020 cells in HIE neonates with hypothermia therapy. CL2020, human muse (multilineage-differentiating stress-enduring) cells, are being used for this study. They are identified as a subpopulation of mesenchymal stem/stromal cells (MSCs), which have the pluripotency to develop into three germ layers. In this study, neonates with HIE undergoing TH will be administered intravenously with 1.5 million or 15 million CL2020 cells on days 5 to 14 of birth. The study’s primary endpoint includes the evaluation of the incidence of adverse events until 12 weeks after the administration.

##### NCT01019733: Intrathecal Stem Cells in Brain Injury (ISC)

This study was designed as a prospective, open-label, non-randomized phase I clinical trial. The study was aimed to evaluate the safety, tolerability, and efficacy of autologous bone marrow-derived total nucleated cell (TNC) intrathecal injection in pediatric patients with cerebral palsy (CP) due to perinatal hypoxic-ischemic injury (1 month to 8 years old). The procedure involved stimulating the bone marrow with granulocyte colony-stimulating factor to obtain TNCs. The intrathecal injection delivered a median of 13.12 × 10^8^ TNCs, including 10.02 × 10^6^ CD34+ cells, while the remaining cells were administered intravenously. Motor, cognitive, communication, personal-social, and adaptive areas were assessed using the Battelle Developmental Inventory at baseline and 1 and 6 months post-procedure. An MRI was performed at baseline and 6 months. Three patients experienced early adverse effects such as headaches, vomiting, fever, and stiff necks, but no serious complications were reported. According to the Battelle Developmental Inventory, the overall developmental age increased by 4.7 months, with no MRI changes observed at 6 months. The study suggests that the subarachnoid placement of autologous bone marrow-derived TNCs in children with cerebral palsy is safe and may improve neurological function.

##### PMID: 37285522: A Pilot Phase I Trial of Allogeneic Umbilical Cord Tissue-Derived Mesenchymal Stromal Cells in Neonates with Hypoxic-Ischemic Encephalopathy

Allogeneic cord tissue mesenchymal stromal cells (hCT-MSC) have been shown to ameliorate brain injury in animal models of HIE. In this pilot phase I clinical trial, the safety and preliminary efficacy of hCT-MSC in neonates with HIE were tested. Infants with moderate to severe HIE were included in the study. They were randomized and treated by TH with 1 or 2 doses of 2 million hCT-MSC/kg, intravenously. A total of six neonates, with four of them having moderate and two of them having severe HIE, were enrolled. Babies received 1 or 2 doses of hCT-MSC. They received the first dose during TH and the second dose after 2 months. The results demonstrated that hCT-MSC infusions were well tolerated, although five out of six babies developed low-titer anti-HLA antibodies by 1 year of age. All babies after treatments survived and had average to low-average developmental scores for ages between 12 and 17 months of age [145].

#### 4.2.8. Sovateltide

Sovateltide (IRL-1620 or PMZ-1620) is a synthetic analog of ET-1 and is a highly specific endothelin B (ETB) receptor agonist [146,147,148,149]. Several studies have demonstrated its beneficial effects on apoptosis, oxidative stress, angiogenesis, neurogenesis, neuronal repair, and regeneration in the brain after injury, leading to improved neurological and motor functions [146,148,149,150,151,152,153,154,155,156,157,158]. We are currently developing sovateltide as a “First-in-Class” therapeutic for treating ischemic stroke as well as neonatal HIE. Animal models of ischemic stroke have shown promising results, including improved hypoxia-induced survival factors, survival rates, reduced neurological and motor deficits, and decreased infarct volume, edema, and oxidative stress in the injured brain [146,149,151,152,153,155,156,157,158,159]. The phase I, II, and III clinical trials have concluded that sovateltide is safe, well-tolerated, and significantly effective in improving neurological outcomes in acute cerebral ischemic stroke patients at 90 days of treatment [160,161]. Because of similar pathophysiology (ischemia/hypoxia) and neural cell damage in ischemic stroke and HIE, sovateltide could be useful in curbing the pathophysiological progression of HIE by controlling the primary and secondary energy failure through increasing hypoxia-induced survival factors and reducing oxidative stress and cell death in the neonatal hypoxic-ischemic brain. Additionally, by promoting angiogenesis and neurogenesis, sovateltide would increase plasticity and reduce neuronal cell loss following HIE (Figure 1).

##### Development of Sovateltide as a “First in Class” Therapeutic for Neonatal HIE

Given the similarities in pathophysiology (ischemia/hypoxia) between ischemic stroke and HIE, sovateltide could potentially be beneficial in curbing the progression of HIE. By modulating hypoxia-induced survival factors, reducing oxidative stress, and promoting angiogenesis and neurogenesis, sovateltide may help mitigate primary and secondary energy failure and reduce neuronal cell loss following HIE. The research on sovateltide’s neuroprotective and neuroregenerative properties and its positive outcomes in ischemic stroke models [137,143,144,146,147,148,149,150] provides a strong rationale for evaluating its efficacy in treating HIE. The evaluation of sovateltide’s effects on ETB receptor expression, VEGF, NGF, oxidative stress markers, and DNA fragmentation in a Rice–Vannucci rat model of HIE has offered valuable insights into its potential as a therapeutic agent for HIE [148,162,163]. Sovateltide administration showed a significant upregulation of ETB receptor expression [148]. This upregulation of ETB receptors likely contributes to the observed neuroprotective effects of sovateltide. ETB receptors play a crucial role in the development of the nervous system, regulating neuronal differentiation, proliferation, and migration in both the peripheral and central nervous systems. Furthermore, sovateltide treatment enhanced VEGF and NGF expression, important factors for neuronal survival, neuroprotection, and regenerative processes such as growth, differentiation, and axonal outgrowth [148]. The increased levels of VEGF indicated enhanced angiogenic remodeling, which may facilitate recovery following HIE. HIE is also associated with oxidative stress, which can lead to cell death [164]. Sovateltide treatment reduced oxidative stress markers, such as increased GSH and SOD activity, and decreased MDA levels [148]. These results suggest that sovateltide may protect against oxygen-free radical generation and reduce cell damage after brain injury. Furthermore, the positive impact of sovateltide on cell damage and cell death, as assessed through the 7AAD assay, supports its potential as a therapeutic agent for improved neuronal recovery after hypoxic-ischemic insult [148]. Thus, the effectiveness of sovateltide alone or as a therapeutic hypothermia (TH) adjuvant in reducing HIE-mediated disability and death has been demonstrated in a preclinical setting.

Recently, Pharmazz filed an application for “Investigational New Drug (IND)” and was granted permission by the Indian Central Drugs Standard Control Organization (CDSCO) to conduct a phase II trial (NCT05514340) of sovateltide in neonates affected by HIE in India.

##### NCT05514340: Assess Safety and Efficacy of Sovateltide in Hypoxic-Ischemic Encephalopathy

This multicenter, randomized, double-blind, placebo-controlled, phase II trial aims to assess the safety and efficacy of sovateltide treatment for HIE in neonates. The study will involve 40 participants. Participants must be either sex with gestational age ≥ 36 weeks, receiving supportive management for perinatal asphyxia, or showing signs of moderate/severe encephalopathy. Informed consent from parents or legal representatives will be obtained before participation. Those with certain conditions, such as TORCH infection, severe dysmorphic features, and microcephaly, will be excluded from the study. The study has two treatment groups—sovateltide and normal saline. In the “sovateltide group”, participants will receive three doses of sovateltide at 0.3 μg/kg body weight as an intravenous bolus over 1 min every 3 h on days 1, 3, and 6. The “normal saline group” will receive three doses of an equal volume of normal saline as an IV bolus over 1 min at the same intervals. Both treatment groups will receive the best available standard of care, and the study will be quadruple-masked, with participants, care providers, investigators, and outcome assessors blinded to the identity of the assigned treatment. The study’s primary purpose is to assess the safety and efficacy of sovateltide in treating hypoxic-ischemic encephalopathy in neonates. The primary outcome measure will be the percentage of patients with death or disability (moderate/severe) at 24 months. Secondary outcome measures include evaluating changes in Bayley Scales of Infant and Toddler Development Scores, the proportion of children with disabling cerebral palsy, the proportion of patients with seizures, brain injury, blindness, or hearing impairment, the incidence of sovateltide-related adverse events, and tolerance to the treatment. The study is estimated to be completed in 2024–2025.

Overall, the findings from the preclinical studies and the approval for conducting phase II trials in neonatal HIE patients in India demonstrate the potential of sovateltide as a neuroprotective and neuroregenerative agent for treating HIE. However, further research and clinical studies will be crucial to fully understand its efficacy and safety in human patients before considering its widespread therapeutic use. Our ongoing efforts to investigate sovateltide’s therapeutic potential offer hope for addressing the critical need for effective treatments for HIE, potentially improving outcomes for HIE-affected neonates.

## 5. Conclusions

Neonatal hypoxic-ischemic encephalopathy (HIE) is a serious condition that arises due to perinatal asphyxia or ischemia and can lead to significant neurological impairments and even death in newborns. At present, TH is the only approved treatment for HIE, which helps in improving outcomes for infants with HIE, but it has serious shortcomings, including the requirement for costly equipment and management, high variability, poor long-term outcomes, and poorly understood mechanisms. Hence, drug development may focus on supporting and enhancing the efficacy of hypothermia as adjuvants or/and identifying effective treatments that can provide neuroprotection and improve long-term outcomes for affected infants. However, because of the complex and multifaceted nature of HIE, successful drug development requires a multimodal approach. Targeting multiple pathways involved in the injury process, such as inflammation, oxidative stress, and apoptosis, may yield more effective therapeutic outcomes. Developing neuroprotective agents that can limit the extent of brain injury after a hypoxic-ischemic event is crucial. These agents should ideally preserve brain tissue, promote neuroregeneration, and minimize secondary damage. Investigating the potential benefits of combining therapeutic agents with hypothermia as adjuvants could be a promising avenue. Numerous drugs are being evaluated as adjuvants to TH. Drugs like erythropoietin, darbepoetin, xenon, topiramate, and glucocorticoids showed promising results in pre-clinical studies; however, none of them have demonstrated a clear indication of efficacy in improving the long-term outcome in large clinical trials on HIE neonates. Some other therapeutic agents, such as allopurinol, melatonin, metformin, caffeine, citicoline, RLS0071, and stem cells, are being examined through various clinical trials as potential future drugs. The potential of sovateltide to be developed as a novel therapeutic agent to treat neonatal HIE with or without TH is being evaluated at Pharmazz. Sovateltide is being developed as a “First-in-Class” neural progenitor inducer-based therapeutic and has proven safety, tolerability, and efficacy in clinical trials conducted on acute ischemic stroke adult patients in India. Moreover, pre-clinical studies in the HIE animal model showed that sovateltide could also be effective in treating neonatal HIE, as it significantly reduced oxidative and hypoxic damage and induced angiogenesis as well as neurogenesis in the injured neonatal brain of the animal with HIE. Currently, a multicenter, randomized, double-blind, placebo-controlled, phase II trial to assess the long-term (24 months of age) safety and efficacy of sovateltide in neonates with HIE receiving SOC (NCT05514340; n = 40) is recruiting patients. Sovateltide has demonstrated the multimodal effects required for containing the hypoxic-ischemic injury and repairing it by inducing neuronal regeneration and function in the brain affected by HIE. It is anticipated that sovateltide and other future therapeutics that have multimodal action, such as stem cells, allopurinol, RLS 0071, etc., would have a higher potential to be developed as effective therapies or TH adjuvants for treating neonatal HIE and reducing mortality as well as neurodevelopmental disorders in the near future. In conclusion, drug development for neonatal HIE is a challenging and evolving field. Finding effective treatments will require a comprehensive understanding of the disease pathophysiology, rigorous preclinical testing, well-designed clinical trials, and a commitment to improving the long-term outcomes of HIE-affected infants.

## Figures and Tables

**Table 1:** List of registered ongoing/future interventional clinical trials for neonatal HIE. Source: ClinicalTrials.gov (accessed on 3 June 2023).

No.	NCT Number	Study Title	Study Status	Conditions	Interventions	Sponsor	Study Type
1	NCT01962233	Umbilical Cord Derived Mesenchymal Stem Cells Therapy in Hypoxic Ischemic Encephalopathy	UNKNOWN	Hypoxic-Ischemic Encephalopathy	BIOLOGICAL: Mesenchymal stem cells	Hebei Medical University	INTERVENTIONAL
2	NCT05610085	A Dose Escalation Study of Levetiracetam in the Treatment of Neonatal Seizures	RECRUITING	Neonatal Seizure|Neonatal Encephalopathy|Hypoxic-Ischemic Encephalopathy|Seizure Newborn	DRUG: Levetiracetam injection|DRUG: Phenobarbital Sodium injection	University of California, San Diego	INTERVENTIONAL
3	NCT04772222	Dexmedetomidine Use in Infants Undergoing Cooling Due to Neonatal Encephalopathy (DICE Trial)	RECRUITING	Hypoxic-Ischemic Encephalopathy|Pain	DRUG: Dexmedetomidine hydrochloride|DRUG: Morphine sulfate	University of Utah	INTERVENTIONAL
4	NCT05687708	Effect of Non-nutritive Sucking on Transition to Oral Feeding in Infants With Asphyxia	RECRUITING	Swallowing Disorder|Perinatal Asphyxia|Feeding; Difficult, Newborn|Feeding Disorder of Infancy or Early Childhood|Hypoxic-Ischemic Encephalopathy|Speech Therapy	OTHER: Non-nutritive sucking	Medipol University	INTERVENTIONAL
5	NCT05851391	RESTORE: buRst-supprESsion TO Stop Refractory Status Epilepticus Post-cardiac Arrest	NOT_YET_RECRUITING	Hypoxia-Ischemia, Brain|Heart Arrest|Status Epilepticus|Refractory Status Epilepticus|Seizures|Anoxic-Ischemic Encephalopathy|Anoxia-Ischemia, Cerebral	DRUG: Burst suppression EEG target intravenous anesthesia|DRUG: Seizure suppression EEG target intravenous anesthesia	University of California, San Francisco	INTERVENTIONAL
6	NCT04063215	A Clinical Trial to Determine the Safety and Efficacy of Hope Biosciences Autologous Mesenchymal Stem Cell Therapy for the Treatment of Traumatic Brain Injury and Hypoxic-Ischemic Encephalopathy	ACTIVE_NOT_RECRUITING	Traumatic Brain Injury	BIOLOGICAL: HB-adMSCs	Hope Biosciences	INTERVENTIONAL
7	NCT03549520	CEUS Evaluation of Hypoxic Ischemic Injury	RECRUITING	Hypoxic-Ischemic Encephalopathy|Brain Ischemia Hypoxia	DRUG: Sulfur hexafluoride lipid-type A microspheres	Children’s Hospital of Philadelphia	INTERVENTIONAL
8	NCT02854579	Neural Progenitor Cell and Paracrine Factors to Treat Hypoxic Ischemic Encephalopathy	UNKNOWN	Hypoxic-Ischemic Encephalopathy	BIOLOGICAL: Neural progenitor cell|BIOLOGICAL: Paracrine factors|BIOLOGICAL: Progenitor cell and paracrine factors	Navy General Hospital, Beijing	INTERVENTIONAL
9	NCT03806816	Use of Melatonin for Neuroprotection in Asphyxiated Newborns	UNKNOWN	Hypoxic-Ischemic Encephalopathy|Cell Damage|Asphyxia Perinatal	DIETARY_SUPPLEMENT: Melatonin|OTHER: Placebo group	University Hospital of Ferrara	INTERVENTIONAL
10	NCT02551003	Neuroprotective Effect of Autologous Cord Blood Combined With Therapeutic Hypothermia Following Neonatal Encephalopathy	RECRUITING	Hypoxic-Ischemic Encephalopathy|Cerebral Infarction	DRUG: Autologous cord blood|DEVICE: Hypothermia	Children’s Hospital of Fudan University	INTERVENTIONAL
11	NCT05039697	Normobaric Hyperoxia Combined With Endovascular Therapy in Patients With Stroke Within 6 Hours of Onsetï¼šLongterm Outcome	UNKNOWN	Stroke, Acute|Hypoxia-Ischemia, Brain|ENDOVASCULAR TREATMENT	OTHER: Oxygen	Capital Medical University	INTERVENTIONAL
12	NCT03079167	PAEAN—Erythropoietin for Hypoxic Ischaemic Encephalopathy in Newborns	ACTIVE_NOT_RECRUITING	Hypoxic-Ischemic Encephalopathy	DRUG: Epoetin alfa|DRUG: Normal saline	University of Sydney	INTERVENTIONAL
13	NCT01646619	Efficacy Study of Hypothermia Plus Magnesium Sulphate(MgSO4) in the Management of Term and Near Term Babies With Hypoxic Ischemic Encephalopathy	UNKNOWN	Severe Hypoxic-Ischemic Encephalopathy|Moderate Hypoxic Ischemic Encephalopathy	DRUG: Magnesium sulphate|DRUG: Placebo	Sajjad Rahman	INTERVENTIONAL
14	NCT03913221	Caffeine for Hypoxic-Ischemic Encephalopathy	ACTIVE_NOT_RECRUITING	Hypoxic-Ischemic Encephalopathy	DRUG: Caffeine citrate 5 mg/kg|DRUG: Caffeine citrate 10 mg/kg	University of North Carolina, Chapel Hill	INTERVENTIONAL
15	NCT03181646	Role of Citicoline in Treatment of Newborns With Hypoxic Ischemic Encephalopathy	UNKNOWN	Hypoxic-Ischemic Encephalopathy	DRUG: Citicoline	Armed Forces Hospital, Pakistan	INTERVENTIONAL
16	NCT04217421	Cerebrum and Cardiac Protection With Allopurinol in Neonates With Critical Congenital Heart Disease Requiring Cardiac Surgery With Cardiopulmonary Bypass	UNKNOWN	Congenital Heart Disease in Children|Neuroprotection	DRUG: Allopurinol|DRUG: Mannitol	dr. M.J.N.L. Benders	INTERVENTIONAL
17	NCT03162653	Effect of Allopurinol for Hypoxic-ischemic Brain Injury on Neurocognitive Outcome	RECRUITING	Encephalopathy, Hypoxic-Ischemic|Infant, Newborn, Diseases	DRUG: Allopurinol|DRUG: Mannitol	University Hospital Tuebingen	INTERVENTIONAL
18	NCT05130528	Therapeutic Intervention Supporting Development From NICU to 6 Months for Infants Post Hypoxic-Ischemic Encephalopathy	RECRUITING	Cerebral Palsy|Hypoxic-Ischemic Encephalopathy	BEHAVIORAL: Sensorimotor intervention	University of Southern California	INTERVENTIONAL
19	NCT05590676	Metformin Treatment in Infants After Perinatal Brain Injury	NOT_YET_RECRUITING	Hypoxic-Ischemic Encephalopathy of Newborn|Premature Birth	DRUG: Metformin hydrochloride	The Hospital for Sick Children	INTERVENTIONAL
20	NCT03123081	Role of Umbilical Cord Milking in the Management of Hypoxic-ischemic Encephalopathy in Neonates	UNKNOWN	Hypoxic-Ischemic Encephalopathy	PROCEDURE: Umbilical cord milking	Jubilee Mission Medical College and Research Institute	INTERVENTIONAL
21	NCT02395276	Hypothermia Therapy in Pediatric Cardiac Intensive Care Unit for Suspected for Brain Injury	UNKNOWN	Congenital Heart Defects|Brain Ischemia|Hypoxia Brain|Child|Hypothermia, Induced	DEVICE: Whole body hypothermia	Sheba Medical Center	INTERVENTIONAL
22	NCT04261335	The Clinical Trial of CL2020 Cells for Neonatal Hypoxic Ischemic Encephalopathy	ACTIVE_NOT_RECRUITING	Hypoxia-Ischemia, Brain	BIOLOGICAL: CL2020 cells	Nagoya University	INTERVENTIONAL
23	NCT05568264	Effects of a Physical Therapy Intervention on Motor Delay in Infants Admitted to a Neonatal Intensive Care Unit	RECRUITING	Motor Delay|Premature Birth|Intraventricular Hemorrhage|Hypoxic-Ischemic Encephalopathy|Bronchopulmonary Dysplasia	OTHER: Physical therapy intervention	Shirley Ryan AbilityLab	INTERVENTIONAL
24	NCT05490173	The Pilot Experimental Study of the Neuroprotective Effects of Exosomes in Extremely Low Birth Weight Infants	NOT_YET_RECRUITING	Premature Birth|Extreme Prematurity|Preterm Intraventricular Hemorrhage|Hypoxia-Ischemia, Cerebral|Neurodevelopmental Disorders	OTHER: Exosomes derived from mesenchymal stromal cells (MSCs)	Federal State Budget Institution Research Center for Obstetrics, Gynecology and Perinatology Ministry of Healthcare	INTERVENTIONAL
25	NCT05836610	Hydrocortisone Therapy Optimization During Hypothermia Treatment in Asphyxiated Neonates	RECRUITING	Hypoxic-Ischemic Encephalopathy|Asphyxia|Hypotension|Circulatory Failure Neonatal	DRUG: Hydrocortisone	Semmelweis University	INTERVENTIONAL
26	NCT05648812	Neonatal Brain Ultrasound With CEUS and Elastography	NOT_YET_RECRUITING	Neonatal Hypoxic-Ischemic Encephalopathy|Neonatal Stroke|Neonatal Encephalopathy, Unspecified	DIAGNOSTIC_TEST: Brain contrast enhanced ultrasound, brain ultrasound elastography|DRUG: Sulfur hexafluoride	Turku University Hospital	INTERVENTIONAL
27	NCT03163589	Erythropoietin in Management of Neonatal Hypoxic Ischemic Encephalopathy	UNKNOWN	Hypoxic-Ischemic Encephalopathy	DRUG: Erythropoietin|DRUG: normal saline	Assiut University	INTERVENTIONAL
28	NCT03657394	Comparative Outcomes Related to Delivery-room Cord Milking In Low-resourced Countries	RECRUITING	Hypoxic-Ischemic Encephalopathy|Birth Asphyxia	OTHER: Umbilical cord milking	Nemours Children’s Clinic	INTERVENTIONAL
29	NCT05951777	Autologous Adipose Derived Mesenchymal Stem Cells for Chronic Traumatic Brain Injury	NOT_YET_RECRUITING	Traumatic Brain Injury	BIOLOGICAL: Autologous HB-adMSCs|DRUG: Normal saline	Hope Biosciences	INTERVENTIONAL
30	NCT03352310	Feasibility and Safety of Umbilical Cord Blood Transfusion in the Treatment of Neonatal Cerebral Ischemia and Anemia	UNKNOWN	Hypoxic-Ischemic Encephalopathy|Hypoxia Neonatal|Cerebral Ischemia of Newborn|Anemia, Neonatal	BIOLOGICAL: Autologous umbilical cord blood (UCB)|PROCEDURE: Standard care	Mononuclear Therapeutics Ltd.	INTERVENTIONAL
31	NCT05778188	A Study to Evaluate the Safety, Tolerability, Pharmacokinetics, and Preliminary Efficacy of RLS-0071 in Newborns With Moderate or Severe Hypoxic-Ischemic Encephalopathy Undergoing Therapeutic Hypothermia	NOT_YET_RECRUITING	Hypoxic-Ischemic Encephalopathy	DRUG: RLS-0071|DRUG: Placebo	ReAlta Life Sciences, Inc.	INTERVENTIONAL
32	NCT04217551	Influence of Cooling Duration on Efficacy in Cardiac Arrest Patients	RECRUITING	Cardiac Arrest, Out-Of-Hospital|Hypothermia, Induced|Hypoxia-Ischemia, Brain	DEVICE: Therapeutic Hypothermia	University of Michigan	INTERVENTIONAL
33	NCT03682042	Comparative Outcomes Related to Delivery-room Cord Milking In Low-resourced Countries Developmental Follow Up	RECRUITING	Hypoxic-Ischemic Encephalopathy|Birth Asphyxia	OTHER: Umbilical cord milking	Nemours Children’s Clinic	INTERVENTIONAL
34	NCT05295784	PK and Safety of Caffeine in Neonates With Hypoxic Ischemic Encephalopathy Receiving Therapeutic Hypothermia	NOT_YET_RECRUITING	Acute Kidney Injury|Hypoxic-Ischemic Encephalopathy|Caffeine	DRUG: Caffeine citrate	University of Arkansas	INTERVENTIONAL
35	NCT02578823	Targeted Temperature Management After In-Hospital Cardiac Arrest	UNKNOWN	Hypoxic-Ischemic Encephalopathy	DEVICE: Arctic SunA^®^|DEVICE: Arcticgela|PROCEDURE: Conventional antipyretic treatment	Asan Medical Center	INTERVENTIONAL
36	NCT01765218	Topiramate in Neonates Receiving Whole Body Cooling for Hypoxic Ischemic Encephalopathy	ACTIVE_NOT_RECRUITING	Hypoxic-Ischemic Encephalopathy	DRUG: Topiramate|DRUG: Placebo	Kristin R Hoffman	INTERVENTIONAL
37	NCT05379218	RIC in HIE: A Safety and Feasibility Trial	RECRUITING	Hypoxic-Ischemic Encephalopathy	DEVICE: Remote ischemic conditioning	The Hospital for Sick Children	INTERVENTIONAL
38	NCT02229123	Levetiracetam Treatment of Neonatal Seizures: Safety and Efficacy Phase II Study	UNKNOWN	Neonatal Seizures	DRUG: Intravenous levetiracetam	University Hospital, Tours	INTERVENTIONAL
39	NCT05820178	tDCS and rTMS in Patients With Early Disorders of Consciousness	NOT_YET_RECRUITING	Disorder of Consciousness|Stroke|Ischemic-hypoxic Encephalopathy	DEVICE: tDCS|DEVICE: rTMS	Xuanwu Hospital, Beijing	INTERVENTIONAL
40	NCT03527498	Evaluation of Long-term Neurodevelopment in Neonatal Encephalopathy by Infant Treadmill	RECRUITING	Hypoxic-Ischemic Encephalopathy|Periventricular Leukomalacia|Intraventricular Hemorrhage|Bilirubin Encephalopathy|Kernicterus|Hypoglycemia, Neonatal|Cerebral Infarction	DEVICE: Baby treadmill|BEHAVIORAL: Physical rehabilitation training	Children’s Hospital of Fudan University	INTERVENTIONAL
41	NCT04913324	Early Virtual Intervention for Infants With CP Following HIE Diagnosis	NOT_YET_RECRUITING	Cerebral Palsy|Hypoxic-Ischemic Encephalopathy|Brain Injuries|Perinatal Hypoxia	BEHAVIORAL: Virtual vare	The Hospital for Sick Children	INTERVENTIONAL
42	NCT04896736	Multisite Tissue Oxygenation Guided Perioperative Care in Cardiac Surgery	RECRUITING	Brain Ischemia Hypoxia|Muscle; Ischemic|Muscle Hypoxia	OTHER: Multisite tissue oxygenation-guided care|OTHER: Usual care	Yale University	INTERVENTIONAL
43	NCT02881970	Neonatal Hypoxic Ischemic Encephalopathy: Safety and Feasibility Study of a Curative Treatment With Autologous Cord Blood Stem Cells	RECRUITING	Neonatal Hypoxic-ischaemic Encephalopathy	DRUG: Autologous cord blood stem cell	Assistance Publique Hopitaux De Marseille	INTERVENTIONAL
44	NCT05390060	Delineating Between Pathophysiologic Phenotypes of Hypoxic-ischemic Brain Injury After Cardiac Arrest	RECRUITING	Hypoxia-Ischemia, Brain	DEVICE: Neuromonitoring	University of British Columbia	INTERVENTIONAL
45	NCT01138176	Whole Body Cooling Using Phase Changing Material	UNKNOWN	Hypoxic-Ischemic Encephalopathy	PROCEDURE: Cooling	Robertson, Nicola, M.D.	INTERVENTIONAL
46	NCT02621944	Melatonin as a Neuroprotective Therapy in Neonates With HIE Undergoing Hypothermia	RECRUITING	Hypoxic-Ischemic Encephalopathy	DRUG: Melatonin|OTHER: Magnetic resonance imaging|OTHER: Pharmacokinetics|BEHAVIORAL: Neurological outcome assessment	University of Florida	INTERVENTIONAL
47	NCT05514340	Assess Safety and Efficacy of Sovateltide in Hypoxic-ischemic Encephalopathy	RECRUITING	Hypoxic-Ischemic Encephalopathy|Neonatal Asphyxia|Neonatal Encephalopathy	DRUG: Normal Saline along with standard treatment|DRUG: Sovateltide along with standard treatment	Pharmazz, Inc.	INTERVENTIONAL

## Data Availability

Not applicable.

## Abbreviations

HIE	Hypoxic-Ischemic Encephalopathy
TH	Therapeutic Hypothermia
ATP	Adenosine Triphosphate
BI	Barthel Index
EQ-5D	European Quality of Life Five Dimension
GP	Glial Progenitors
IV	Intravenous
mRS	Modified Rankin Scale
NICU	Neonatal Intensive Care Unit
NIHSS	National Institutes of Health Stroke Scale
SSQoL	Stroke Specific Quality of Life Scale
VEGF	Vascular Endothelial Growth Factor
NGF	Nerve Growth Factor
Body wt.	Body weight
MRI	Magnetic Resonance Imaging
GABA	Gamma Aminobutyric Acid
ACD	Accidental Cell Death
PCD	Programmed Cell Death
ROS	Reactive Oxygen Species
RNS	Reactive Nitrogen Species
NOS	Nitric Oxide Synthase
COX	Cyclooxygenases
XO	Xanthine Oxidase
PTP	Protein Tyrosine Phosphatase
TK	Tyrosine Kinase
FOXO	Forkhead Box O
HIF	Hypoxia Inducible Factor
EEG	Electroencephalography
aEEG	amplitude integrated EEG
NMDA	N-methyl-D-aspartate
KATP	ATP-sensitive Potassium Channels
K(2P)	Two-pore Domain Potassium Channels
EPO	Erythropoietin
SAEs	Serious Adverse Events
SOC	Standard of Care
USFDA	United States Food and Drug Administration
CNS	Central Nervous System
NCT	National Clinical Trial
BBB	Blood–Brain Barrier
MSCs	Mesenchymal Stem Cells
ET-1	Endothelin 1
GSH	Glutathione
SOD	Superoxide Dismutase
MDA	Malondialdehyde
